# IDO2 in Immunomodulation and Autoimmune Disease

**DOI:** 10.3389/fimmu.2014.00585

**Published:** 2014-11-20

**Authors:** George C. Prendergast, Richard Metz, Alexander J. Muller, Lauren M. F. Merlo, Laura Mandik-Nayak

**Affiliations:** ^1^Lankenau Institute for Medical Research, Wynnewood, PA, USA; ^2^Department of Pathology, Anatomy and Cell Biology, Jefferson Medical School, Thomas Jefferson University, Philadelphia, PA, USA; ^3^Kimmel Cancer Center, Thomas Jefferson University, Philadelphia, PA, USA; ^4^New Link Genetics Corporation, Ames, IA, USA; ^5^Department of Microbiology and Immunology, Thomas Jefferson University, Philadelphia, PA, USA

**Keywords:** indoleamine dioxygenase, tolerance, kynurenine pathway, aryl hydrocarbon receptor, autoimmunity, rheumatoid arthritis

## Abstract

IDO2 is a relative of IDO1 implicated in tryptophan catabolism and immune modulation but its specific contributions to normal physiology and pathophysiology are not known. Evolutionary genetic studies suggest that IDO2 has a unique function ancestral to IDO1. In mice, IDO2 gene deletion does not appreciably affect embryonic development or hematopoiesis, but it leads to defects in allergic or autoimmune responses and in the ability of IDO1 to influence the generation of T regulatory cells. Gene expression studies indicate that IDO2 is a basally and more narrowly expressed gene than IDO1 and that IDO2 is uniquely regulated by AhR, which serves as a physiological receptor for the tryptophan catabolite kynurenine. In the established KRN transgenic mouse model of rheumatoid arthritis, where IDO1 gene deletion has no effect, IDO2 deletion selectively blunts responses to autoantigen but has no effect on responses to neoantigen challenge. In human populations, natural variations in IDO2 gene sequence that attenuate enzymatic activity have been reported to influence brain cancer control and adaptive immune responses to the IDO2 protein itself, consistent with the concept that IDO2 is involved in shaping immune tolerance in human beings. Biochemical and pharmacological studies provide further evidence of differences in IDO2 enzymology and function relative to IDO1. We suggest that IDO2 may act in a distinct manner from IDO1 as a set-point for tolerance to “altered-self” antigens along the self-non-self continuum where immune challenges from cancer and autoimmunity may arise.

## Introduction

Of the four tryptophan catabolic enzymes in mammals, IDO2 is the most recently discovered and like the others (IDO1, TDO2, and TPH1), it is implicated in immune control. While all the three dioxygenases in this group (IDO1, IDO2, and TDO2) generate kynurenine as a product, TDO2 represents a structurally distinct multimeric enzyme of divergent origin compared to the monomeric IDO1 and IDO2 enzymes, which are related. The human *IDO2* gene is located downstream of *IDO1* on chromosome 8p21 and these two genes bear close structural and evolutionary relationships. Compared to the other enzymes, IDO2 expression is confined mainly to antigen-presenting immune cells, liver, kidney, brain, and placenta, displaying a unique and relatively more restricted pattern that is consistent with a non-redundant function(s). Early studies of the physiological function of IDO1 by Munn, Mellor, and colleagues pioneered the concept that tryptophan catabolism modulates immunity, based on the discovery that a simple tryptophan mimetic, the IDO inhibitor D,L-1-methyl-tryptophan (1MT), could trigger rejection of allogeneic murine concept (1, 2). Subsequent to this discovery, 1MT has been used in thousands of studies to study IDO function in diverse settings of immune control. However, interpreting these studies may be impacted by the discovery of IDO2, which under various conditions has been found to be inhibited by 1MT like IDO1 (3–7). Thus, while 1MT has been used widely to implicate tryptophan catabolism in numerous chronic inflammatory pathologies, such as cancer, chronic infection, allergy, neurological disorders, and autoimmunity (8, 9), the possible contributions of IDO2 in interpreting the effects of 1MT may be impactful. Another fine review on IDO2 has appeared recently (10). This review summarizes existing knowledge about IDO2 and its functions in immune control and disease.

## IDO2 Discovery

IDO2 was discovered independently by groups working in the areas of infectious disease, cancer research, and genomics (3, 11, 12). Ball et al. cloned IDO2 by searching cDNA libraries used in high-throughput sequencing for IDO1-like sequences, identifying in this manner a novel gene they termed INDOL1 (11). Recombinant enzyme was shown to catabolize tryptophan to kynurenine like IDO1 but with a reduced relative activity. Comparative genomics provided evidence that IDO2 arose by gene duplication before the origin of the tetrapods. Expression was documented in kidney, liver, and epididymis, localizing the endogenous IDO2 enzyme to kidney tubular cells and spermatozoa. Distinct functions were suggested by differences in the catalytic expression patterns noted within tissues and during malaria infection.

Metz et al. cloned IDO2 on the basis of partial IDO1 structural homologies that were found downstream of the human IDO1 gene in a region of chromosome 8p12 that was misannotated in early genome compilations (3). This work documented the tryptophan catalytic activity of mouse and human cDNAs, with the mouse isoform exhibiting higher catabolic activity but both isoforms showing less activity compared to IDO1 under similar conditions. Two single nucleotide polymorphisms (SNP) were described in the IDO2 coding region that were widely distributed in human populations, R248W and Y359X, each of which attenuated catalytic activity. A narrow range of IDO2 expression was documented by mouse tissue analysis with highest expression in liver, kidney, and placenta. Complex RNA splicing patterns were revealed in placenta and brain. In human, 293 cells engineered to overexpress IDO1 and IDO2, there were differences in how tryptophan depletion mediated by each enzyme affected translation by regulating eIF2α, with IDO2-expressing cells unresponsive to subsequent tryptophan restoration suggestive of a pseudo-differentiation effect. Moreover, IDO2-expressing cells exhibited a unique susceptibility to catalytic inhibition by the D isoform of 1MT, which selectively impeded the activity of full-length IDO2 but not IDO1 in this setting (3).

Yuasa et al. described a novel mouse IDO2 cDNA identified as an IDO1 paralog in a set of several evolutionary studies of IDO genes that supported the concept of IDO2 functional differences (12). Characterizing the activity of the recombinant mouse enzyme, they noted the relatively lower tryptophan catabolic activity of IDO2 compared to IDO1-like Ball et al. and Metz et al. Based on a phylogenetic analysis, they argued that IDO2 and other low-activity IDO paralogs from non-mammalian organisms were proto-IDO enzymes (12). Interestingly, while IDO-like genes were observed in several lower vertebrates, the genomes from chicken and zebrafish exhibited only one IDO-related gene most similar to IDO2. Accordingly, they argued that IDO1 may have arisen by gene duplication of a more ancient proto-IDO gene before the divergence of marsupial and eutherian (placental) mammals. Given the relatively weaker catalytic activity of IDO2 enzymes, this group suggested that L-Trp may not be a true *in vivo* physiological substrate, although methylene blue rather than physiological reductants (used by all groups in the oxygenase reactions studied) might not provide reliable insights into function, as noted in Ref. (6). This intriguing suggestion is consistent with the finding of Metz et al., who found that IDO2-overexpressing human 293 cells were unresponsive to tryptophan restoration after tryptophan had been depleted in cell culture by IDO2 activity, in stark contrast to IDO1-overexpressing cells, which responded fully.

A subsequent study of fungal IDO homologs by this group further corroborated the hypothesis that IDO2 functions in some unique manner (13). Specifically, this work revealed that the tryptophan catabolic activity of some fungal IDO enzymes was sufficient to supply nicotinamide adenine dinucleotide (NAD), the downstream end product of the kynurenine pathway, whereas other fungal IDO enzymes lacked sufficient tryptophan catabolic activity to supply NAD. Thus, it seems clear that low catalytic efficiency IDO enzymes are only conserved in evolution, but that they also diverged from active IDO enzymes at early times. Overall, initial characterization of IDO2 suggested features arguing for a unique functional role(s) relative to IDO1.

## IDO2 Expression Patterns

Several studies have described expression patterns of IDO2 message and protein that suggest unique regulation but also some functional redundancy with IDO1. Whereas IDO1 predominates in colon and epididymis, IDO2 mRNA predominates in cerebral cortex, liver, and kidney. Evidence of redundancy is suggested by the finding that *Ido1* genetic deficiency in mice leads to compensatory upregulation of IDO2 in the epididymis, where IDO1 is relatively more highly expressed normally (14). IDO2 is also expressed like IDO1 in antigen-presenting cells but under somewhat different control. The IDO2 promoter includes a prominent binding site for the transcription factor IRF-7, a master regulator of dendritic cell maturation, suggesting a central role in these professional antigen-presenting cells (15). In this setting, IDO2 appears to be a mainly basally expressed gene, the levels of which vary little by comparison to IDO1 levels that are more robustly regulated. Current information suggests that at the RNA level IDO2 expression is regulated by various pro-inflammatory stimuli, but less robustly than IDO1, including in dendritic cells by interferon-γ (IFN-γ), IL-10, lipopolysaccharide, and prostaglandin E2 (3, 15–18). Interestingly, activation of the aryl hydrocarbon receptor (AhR), a transcription factor, which can serve as a physiological ligand for kynurenine (19), has been reported to upregulate IDO2 in dendritic cells (20, 21). Since activated IDO1 generates kynurenine, this observation presents the intriguing possibility of a downstream mechanism to elevate IDO2 levels in dendritic cells where IDO1 becomes upregulated, a prospect discussed further below. IDO2 has been reported to be overexpressed along with IDO1 in pancreatic cancer (22, 23), and in basal cell skin carcinomas, where its expression appears to be driven by the T-cell-attracting chemokine CXCL11 (16), but neither the extent of IDO2 expression nor knowledge of its regulatory mechanisms in cancer settings are as widely described as IDO1 as yet.

## Mouse Genetic Studies: IDO2 is Critical for IDO1-Dependent Treg Generation

Our group constructed and characterized mice that are genetically deficient in *Ido2* to investigate its functions in development, normal physiology, and pathophysiology (24). These mice retain the normal structure and expression of the nearby upstream *Ido1* gene. Interest in generating this strain was reinforced by our discovery that IDO2 RNA levels were attenuated in myeloid cells from *Ido1*^−/−^ mice due to an altered RNA splicing event that abolishes catalytic function (24). This was a tissue-specific effect insofar as IDO2 RNA splicing was unaffected in livers from *Ido1*^−/−^ mice. How IDO1 may affect IDO2 RNA processing was unclear but likely to be indirect. Nevertheless, it appeared that in addition to their deficiency in IDO1 function *Ido1*^−/−^ mice were also mosaic deficient for IDO2 function. This revelation was important since it influences the interpretation of phenotypic results involving myeloid cells from *Ido1*^−/−^ mice, widely studied in the field, which might conceivably be explained by loss of function in IDO2 rather than IDO1. Indeed, the possibility of an IDO1 → IDO2 genetic pathway in myeloid cells was consistent with expression data from Bankati et al., who had found that activation of the kynurenine-stimulated transcription factor AhR was sufficient to stimulate IDO2 transcription (21). Overall, we reasoned that mice deficient in IDO2 might not only help define its functions but also help re-interpret of functions previously ascribed to IDO1 (made on the basis of findings from *Ido1*^−/−^ mice).

IDO1 acts to control the activation and differentiation of T regulatory cells (Treg) in a variety of settings, including cancer (25–27). Given evidence of genetic epistasis between IDO1 and IDO2, we asked whether IDO2 loss could affect IDO1-mediated Treg generation in settings where an essential function for IDO1 has been established. In WT or *Ido2*^−/−^ mice treated with CpG oligonucleotides, a critical role for IDO2 in Treg generation was documented in an established T-cell suppression assay (26). Suppression relieved by *Ido2* loss in this assay was reversed by a cocktail of PD1 and PD-L1/PD-L2 antibodies that block PD-1 interaction with PD-L1/PD-L2, a hallmark of IDO1-activated Tregs (26). Strikingly, the effect observed phenocopied the effects of *Ido1* loss in Treg cells generated under the same conditions, directly supporting a functional requirement for IDO2 in Treg generation and offering further evidence of its genetic epistatic interaction with IDO1.

Comparing the response of *Ido2*-deficient mice in a classical assay for contact hypersensitivity led to further support for a role of IDO2 in T-cell-dependent immune responses (24). While *Ido1*^−/−^ and *Ido2*^−/−^ mice both displayed a reduction in contact hypersensitivity, relative to WT control animals, loss of *Ido2* but not *Ido1* was associated with a reduction in systemic levels of cytokines implicated causally in this classical immune response (GM-CSF, G-CSF, IFN-γ, TNF-α, IL-6, and CCL2). Reductions in GM-CSF might be relevant in skin, given its critical role in stimulating AhR-dependent maturation of Langerhans cells (LC) (28), which are thought to be involved in skin tolerance. Since IDO2 is itself an AhR target gene (20, 21), one plausible model is that IDO2 acts downstream of AhR to support local expression of GM-CSF, thereby promoting LC maturation and LC-mediated tolerance through an autocrine loop. While focused mechanistic investigations are needed such models may offer a logical starting point to interpret how IDO2 may act in antigen-presenting cells to influence T-cell function.

Distinct contributions to pathogenic inflammatory processes were likewise indicated in skin carcinogenesis assays, where tumors are induced by a single topical administration of the Ras mutagen DMBA followed by chronic weekly exposure to the pro-inflammatory phorbol ester TPA. Here, while *Ido1* loss was sufficient to blunt tumor formation, as observed previously (29), *Ido2* loss had no effect on the susceptibility to either formation or progression of tumors (24). Taken together, these results offered the first direct physiological evidence that IDO2 helps regulate adaptive immunity, perhaps through contributions to inflammatory control that are at least partly non-redundant with IDO1.

## Mouse Genetic Studies: IDO2 is Critical for Autoantibody Production and Autoimmunity

Rheumatoid arthritis (RA) is an autoimmune disorder that has been associated with aberrant IDO activity and defective T-cell function (30–32). In IDO studies conducted in preclinical mouse models of RA, there is complexity in interpreting the contributions of IDO to the disease state, with opposing effects depending on the model used. Nevertheless, as an initial assessment of the possible connections between IDO2 and T-cell function in a genetically defined model, we compared the effects of genetic deletion of IDO1 or IDO2 in the KRN transgenic mouse model of spontaneous RA. The specific pathophysiological relevancy of the KRN model to human disease is justified in part by its mimicry of the elevated tryptophan degradation in RA patients, which has been appreciated clinically for many years [as summarized in Ref. (33)].

In the KRN model of spontaneous RA, we found that IDO2 was crucial for the development of arthritis but that IDO1 was completely dispensable. This finding was provocative in light of earlier observations that D-1MT treatment could attenuate RA in this model (34) and that D-1MT was capable of inhibiting the tryptophan catabolic activity of IDO2 but not IDO1 in human cells (3). Interestingly, while *Ido2* deficiency phenocopied D-1MT treatment, *Ido1* deficiency abolished responses to D-1MT even though *Ido1* was dispensable for RA pathogenicity, providing further support for IDO1-IDO2 genetic interaction in immune control.

Investigations of cellular mechanisms revealed that the decreased joint inflammation displayed by *Ido2*^−/−^ mice relative to control animals was due to a reduction in pathogenic autoantibodies and antibody-secreting B cells. Strikingly, reduced inflammation in *Ido2*^−/−^ mice was associated with a defect in the initiation of autoreactive B cell responses, but not with any overall defect in normal B cell responses: total serum immunoglobulin levels were unaffected in *Ido2*^−/−^ mice, and those mice were fully competent to mount productive antibody responses to model antigens *in vitro* and *in vivo*. *Ido2* deficiency also reduced CD4^+^ helper T-cell responses; however, in this case reciprocal adoptive cell transfer studies showed that this defect was extrinsic to T cells. While interpretation of these results must be tempered in light of distinct effects of IDO signaling in collagen-induced models of arthritis (35), a different preclinical model used in the field, our genetic studies of IDO2 in the mouse nevertheless offer the first direct evidence that it makes unique contributions to the control of adaptive immunity and inflammatory disease.

## IDO2 in Human Studies

In human immune physiology, the implication of a genetic linkage between IDO1 and IDO2 is intriguing in light of the broad distribution of two functionally attenuating SNP in the coding region of the IDO2 gene in human populations (3). These SNP variations dramatically reduce or abolish tryptophan catabolic activity, therefore varying the level of this IDO2 function in different individuals, perhaps affecting T-cell-dependent immune control as a result. Since antigen-presenting cells are a primary site of IDO2 expression, further investigation is needed to understand how IDO2 may act to initiate, maintain, fix, or reverse antigen tolerance. Along these lines, a recent study in human DC suggests that IDO2 may help fix basal levels of tolerance, acting differently than IDO1, which unlike IDO2 is induced strongly by prostaglandin E2 (PGE2) and other pro-inflammatory signals in these cells (15). This study compared the patterns of expression and regulation of IDO1 and IDO2 in human circulating DC. At the protein level, IDO1 was expressed only in circulating myeloid DC and was modulated by PGE2, whereas IDO2 was expressed in both mDC and plasmacytoid DC and was not modulated by PGE2. In circulating DC from healthy subjects, IDO1 expression relied on PGE2 whereas IDO2 expression was constitutive. However, in DC from arthritis patients, circulating DC expressed both IDO1 and IDO2. Notably, mDC and plasmacytoid DC both generated T regulatory cells through a mechanism that relied upon both IDO1 and IDO2 expression, based on the interpretation of RNAi-mediated gene silencing experiments (15). These observations further supported a model for IDO1-IDO2 genetic interaction in antigen-presenting cells. Further, they suggested that IDO2 may act as a downstream basal function in determining “set points” for tolerance determined by IDO1 acting as an upstream inducible function. In any case, this work established that IDO2 is expressed stably in DC under steady-state conditions and that it may contribute to the homeostatic tolerogenic capacity of DC.

The relatively small number of studies of IDO2 in human systems has focused on cancer and where roles in immunosuppression have been hypothesized. In evaluating the use of IDO2 SNP as biomarkers for therapeutic response, Eldredge et al. stratified the response of brain metastasis patients to whole brain radiotherapy (WBRT) when they were orally administered low-dose chloroquine concomitant with therapy (36). This experiment was based on a multipronged rationale. First, patients with brain malignancy who received WBRT were in some cases found to benefit significantly from low-dose chloroquine when administered concomitant with therapy (37). Second, low-dose chloroquine was serendipitously discovered to indirectly inhibit the tryptophan catabolic activity of IDO2 but not IDO1 in cells, apparently by selective interference with the physiologic reductant used by each enzyme in the oxygenase reaction (R. Metz, unpublished observations). Third, the possibility that IDO2 might contribute to cancer immunosuppression in some settings where it is expressed, including brain, similar to the manner in which IDO1 has been implicated widely (9). Fourth, that IDO2 inhibition might relieve immunosuppression in such settings, but only in patients with a functionally active SNP configuration in their IDO2 genes. In a prospective, single-cohort study, WBRT (37.5 Gy in 2.5 Gy daily fractions) administered with concurrent CQ (p.o. 250 mg daily) was safely tolerated in patients with newly diagnosed brain metastases from biopsy-proven, primary lung, breast, or ovarian tumors (*n* = 20). The main finding of this study was a trend toward increased overall survival in patients with wild-type *IDO2* compared to patients with heterozygous or homozygous SNP configurations that ablate *IDO2* enzyme activity (10.4 vs. 4.1 months; *p* = 0.07). In light of evidence that tryptophan catabolism in the brain may influence affective disorders (i.e., mood), it is interesting that a recent study also showed a trend in association of IDO2 region SNP in predicting outcomes to treatment with the anti-depressant drug citalopram (Celexa^®^) (38).

Additional studies encourage the notion that IDO2 may offer some pathogenic support to advanced cancer in certain settings, albeit less widely than IDO1. In cancer, there are reports of IDO2 overexpression in certain gastrointestinal tumors (39), including frequent overexpression in pancreatic cancer (23). Although there is little exploration of this direction as yet, one study reported that skin administration of IDO2 siRNA was as efficient as IDO1 siRNA in promoting the efficacy of a HER2-based DNA vaccine, in a mouse model of breast cancer (40). In a different and more provocative direction, Sorensen et al. have described naturally occurring anti-IDO2 immune responses in the peripheral blood of both cancer patients and healthy donors, specifically, in the presence of a spontaneous cytotoxic T-cell reactivity directed against the IDO2 protein that can recognize and destroy human tumor cells (39). This work extends an earlier description from the same group of a similar parallel response directed against the IDO1 protein (41). More recent work on the IDO2 response stratified the number of responses based on the IDO2 coding region SNP, highlighting stronger responses to homozygous Y359 alleles that do not truncate the IDO2 protein, and more numerous responses to homozygous 248W alleles that reduce tryptophan catabolic activity relative to the wild-type 248R configuration (42). Thus, IDO2 SNP allelic status, which affects tryptophan catalytic function, appears to influence a self-reactive cytotoxic T-cell-dependent response that is directed against IDO2 protein. The role of IDO1 and IDO2 in cytotoxic T-cell responses directed against self has been reviewed recently with a perspective on regulating this response in the setting of cancer therapy (43).

## IDO2 Biochemistry and Pharmacology

Much of the existing literature on IDO2 relates to questions about its biochemical and signaling properties and a budding interest in its pharmacologic inhibition for therapeutic purpose. As alluded to above, there is a general consensus that the tryptophan catabolic activity of IDO2 and IDO2-like genes in non-mammalian organisms is much weaker than IDO1. Studies of the mouse IDO2 enzyme show it to be more catalytically active than the human enzyme, which is quite weak indeed, yet even in mice genetic knockout does not affect systemic kynurenine levels (i.e., as measured in blood serum) (24). Moreover, in cells where IDO2 or IDO1 are overexpressed to levels that deplete tryptophan, inducing autophagy as a result (44), restoring tryptophan is insufficient to relieve protein translation blockades as monitored by a reversal in the expression of the translation stress-induced transcription factor LIP (3, 44), a pathway with pathophysiologic relevance to IDO-driven cancer (45). All in all, the work to date has fed skepticism that tryptophan is an important physiological substrate for IDO2, as Yuasa et al. originally speculated (6). Supporting this idea, three enzymologic studies comparing tryptophan-like compounds as substrates and inhibitors have presented results arguing that human IDO2 may be somewhat more promiscuous than IDO1 (46–48). A screen of a library of FDA-approved drugs for inhibitory activity against recombinant IDO2 identified the proton pump drug tenatoprazole as a low-micromolar inhibitor (IC50 = 1.8 μM), with no IDO1 or TDO2 inhibition up to 50-fold higher drug levels (47). A comparison of recombinant proteins extending the enzymological analysis of human IDO2 reinforced its distinct nature from IDO1, in terms of substrate specificity and affinity, and also based on the identification of tryptophan derivatives that are mutually exclusive as substrates (48). Two groups conducting modeling and experimental testing of novel IDO1 inhibitors demonstrated selectivity against murine IDO2, adding to the evidence of enzymologic differences between IDO1 and IDO2 (49, 50). Going forward, pressing questions to resolve are whether and where tryptophan may be physiologically relevant as an IDO2 substrate, if at all, and whether there are non-tryptophan substrates that are physiologically or pathophysiologically relevant to IDO2 function, as a growing number of investigators seem to currently suspect.

Several studies have addressed the inhibitory properties of 1MT as an IDO2 inhibitor (5–7, 46, 48, 51), particularly with regard to the racemic selectivity of D-1MT in whole cells as originally reported by our group (3). This area of investigation continues to be fraught not only with concerns about suitable physiological substrates, as noted above, but also about suitable physiological reductants involved in the oxygenase reaction(s) that can be mediated by IDO2. We have discussed this issue recently at some length elsewhere (9). Briefly, we have argued that use of methylene blue as a non-physiological reductant obscures the core challenge of how to interpret the ability of 1MT racemers to inhibit IDO2 activity in cells where it may make relevant contributions to normal physiology or pathophysiology, for example, in cancer or autoimmunity. In exploring other reductants, Austin et al. employed cytochrome *b* in oxygenase reactions of recombinant IDO2 but still found it less active than IDO1 and poorly inhibited by either 1MT racemer (46). In examining IDO2 contributions in a human T-cell system, Qian et al. reported that IDO2 could suppress cell growth but that neither 1MT racemer exhibited potency in inhibiting this effect (4). In *Ido2-*deficient mice, we found that genetic ablation of IDO2 but not IDO1 could phenocopy the effect of D-1MT in the context of the KRN model of RA autoimmunity (33, 34). This system offers a murine setting where biochemical studies can be connected to a genetic and pathobiological context, perhaps encouraging investigations in a human setting that can rule in or rule out the relevancy of 1MT as an IDO2 inhibitor. In closing, we propose that the use of IDO2-deficient mice will be useful to advance studies of how immunometabolism mediates tolerance in normal physiology and disease; to gain mechanistic insights into how IDO pathways direct pathogenic inflammation in diverse settings; and to help inform clinical development of IDO and TDO inhibitors being developed to treat cancer and other inflammatory disorders, where early clinical trials have suggested therapeutic promise.

## Conflict of Interest Statement

George C. Prendergast, Richard Metz, and Alexander J. Muller state a conflict of interest as shareholders and George C. Prendergast is also a grant recipient and a member of the scientific advisory board for New Link Genetics Inc., the company that licensed IDO intellectual property for clinical development from the Lankenau Institute of Medical Research, as described in U.S. Patents Nos. 7705022, 7714139, 8008281, 8058416, 8383613, 8389568, 8436151, 8476454 and 8586636. The other authors state no conflict of interest.
